# N-glycosylation enzyme Mpi is essential for mucin O-glycosylation, host-microbe homeostasis, Paneth cell defense, and metabolism

**DOI:** 10.21203/rs.3.rs-6222474/v1

**Published:** 2025-03-25

**Authors:** Avishek Roy, Steve Meregini, Hye-Jeong Cho, Zhenglan Chen, Aariz Zaki, Tandav Argula, Bruce Beutler, Jeffrey A SoRelle

**Affiliations:** 1. Department of Pathology, University of Texas Southwestern Medical Center, Dallas, TX; 2. Center for Genetics of Host Defense, University of Texas Southwestern Medical Center, Dallas, TX; 3. Department of Pediatrics, Division of Allergy and Immunology, University of Texas Southwestern Medical Center, Dallas, TX

**Keywords:** Glycosylation, colitis, mannose, genetic, microbiome, mucus, metabolism

## Abstract

Intestinal homeostasis relies on a protective mucus layer that separates bacteria from the host, with Muc2 as its primary component. This secreted, gel-forming mucin is heavily O-glycosylated, allowing it to retain water and support beneficial bacteria. For the first time, we demonstrate that Muc2 N-glycosylation plays a critical in mucin maturation, O-glycosylation, barrier integrity, and the prevention of dysbiosis. Using mouse models with global and intestine-specific N-glycan deficiency- caused by the loss of the mannose producing enzyme, *Mpi-* we uncover an unexpected link between N-glycosylation and intestinal homeostasis. Our findings reveal that *Mpi* hypomorphic mice are highly sensitive to DSS-induced colitis, while *Mpi*^flox^; *Villin*^Cre^ mice spontaneously develop disease, exhibiting increased ER stress and dysbiosis. Additionally, electron microscopy, proteomics, and gene expression analyses of goblet and Paneth cells indicate immaturity, mitochondrial loss, and disruptions in lipid metabolism. These results highlight the fundamental role of N-glycosylation in maintaining intestinal homeostasis.

## Introduction

Glycosylation is a significant post-translation modification involving the addition of sugar molecules to macromolecules namely-proteins^1^. Over 50% of the proteins involved in regulation of important cellular processes are glycosylated^2^. Mucins are one of the important classes of heavily glycosylated proteins lining the mucosal surfaces including gut epithelium thereby providing a protective barrier^3^. The role of the mucins in the gut is not only restricted for their protective function but also impact the dynamics of the changing microbiome owing to the mucus-bacterial symbiotic relationship^4^. Glycans are added to mucin proteins by O- and N-linked glycosylation in the endoplasmic reticulum and Golgi apparatus essential for its synthesis, maturation and secretory activity^5^. One of the prominent O-glycosylated family of mucins lining gut epithelium is MUC2 and glycosylation status determines the gel-forming property of MUC2 in the form of mucus is essential for the gut protective barrier^6^. Mucus lining the gut also serves as a nutrient source for the commensal bacteria harboring the natural microbiome^7^. The presence of this post translational modification primarily in surface and secreted protein, which are involved in many aspects of physiology; hence, modulations in glycosylation may affect many physiologic and disease process including inflammation, cancer, immunoglobulin therapeutics, and cardiovascular disease^8^ .One of the most prominent health concern originating from deregulation of glycosylation- Congenital disorders of glycosylation (CDG) have provided significant mechanistic insights into systemic, physiologic roles performed by glycans^9^. Mannose phosphate isomerase (*MPI*) involved in the conversion of fructose-6-phosphate (from glycolysis metabolic pathway) to mannose-6-phosphate (M6P) (DEVLIN, T., 2002. Textbook of biochemistry with clinical correlations). This synthesis is the rate-limiting step in mannose synthesis (essential for N-linked glycosylation)^10^. In humans MPI deficiency leads to a clinically identified disorder (CDG-1b= MPI-CDG); it is extremely rare (<1:1,000,000) and characterized by protein-losing enteropathy (PLE), chronic diarrhea, cirrhosis, cyclic vomiting, anemia, and hyperinsulinemic hypoglycemia. This disorder can be corrected using mannose supplementation. Mannose being critical for the core structure in N-linked glycosylation and *MPI* serving as a bottleneck step for mannose synthesis. *MPI* mediated mannose synthesis step could be bypassed by exogenous mannose and allows rescue experiments.

In this study we have generated a first-time tissue specific *MPI* KO mice named *Villin Cre* to understand the effect of glycosylation in intestinal disorders. The intestinal specific KO exhibits spontaneous colitis with symptoms that replicate Ulcerative colitis in humans. Also, *MPI* was found to be involved in the synthesis and maturation of *MUC2* that regulates the mucus secretion lining gut epithelium. This leads to compromised gut protective barrier and dysbiosis. We also observed that Mpi phenotype could be linked to metabolism status owing to the lesser abundance of the metabolic and fatty acid synthesis proteins in the intestinal tissue lysate which could open many questions pertaining to metabolism and its role in the inflammatory bowel disease progression wrt *MPI*. Furthermore, since the benadryl strain displayed reversal of intestinal physiological disorders simply by administration of Mannose thereby it could provide with the opportunities to explore the possibility of glycoengineering for therapeutic purposes.

## Methods

### Mice

A C57BL/6 male is mutagenized with E-Nitroso-N-ethylurea (ENU) and bred to produce G1 founder males.(SoRelle et al., 2020; Wang et al., 2015) G1 males are crossed with C57BL/6J females to create the G2 generation. Recessive mutations can be found in the G3 generation by a backcross of G2 females with their G1 father. The candidate Mpi mutation (p.H54R) was knocked-in as described elsewhere (Lin et al, submitted supplement). The *benadryl* allele was isolated by crossing C1 (1^st^ generation CRISPR mice) to C57BL6/N mice then intercrossing the C2 generation to test C3 mice with the homozygous *benadryl* mutation. The Mpi-flox allele was created with flanking loxP sites around the 3^rd^ *Mpi* exon as described elsewhere (Lin et al, submitted supplement). *Villin-*Cre (Vil1-Cre, JAX stock# 004586) was obtained from Jackson labs. Mice 6 to 16 weeks of age were used in these studies.

### Western Blot

Tissues were lysed by sonication in NP-40 lysis buffer with protease phosphatase inhibitor cocktail (Company), normalized to equivalent total protein levels (30ug) using BCA Kit (Thermo Fisher Scientific) and separated by electrophoresis (Nupage 4–12% Tris gel)(Invitrogen). Antibodies from Cell Signaling Technology (Table No.1), Thermo Fisher Scientific (Table No.1), and MyBioSource (4G9-B4-B8) were used to detect MUC2, from colon and small intestine murine tissue samples of Mpi^F/F^and Mpi^ΔVillin^.

### qPCR

Trizol^®^ extraction of murine tissue in the (Bioruptor Pico Sonication device -Diagenode) yielded pure total RNA used for qPCR experiments. qPCR was performed directly using the mRNA template by Synthesis Kit). Pre-designed primers for Mpi exon 4–5 were purchased from IDT (Mm.PT.58.6004801) and 500uM forward and reverse primers were used with SYBR green master mix and GAPDH Primetime qPCR primers (Mm.PT.39a.1, IDT) were used to calculate the ΔΔCt value of Mpi in mutant and wild type samples. Expression data were normalized to GAPDH as described previously^11^.

### Hematology analysis

EDTA anti-coagulated blood was collected from the facial vein was measured for complete blood count by the HemaVet 950FS (Drew Scientific), which provided red blood cell count, size characteristics, and white blood cell count with differential. Reticulocytes were measured by staining blood cells with thiazole orange (1ng/mL 30 minutes) and anti-CD71 (APC, RI7217, Biolegend)^12^.

### Histology

Tissues were fixed in 4% paraformaldehyde for at least 48 hours then paraffin embedded, sectioned at 5 um, and stained by hematoxylin and eosin, trichrome, periodic acid Schiff, or Alcian blue techniques by the histology core^13^.

### DSS treatment

2–3% DSS (Thermo Scientific) was used in drinking water for 5–7 days to induce colitis in the Mpi^ben/ben^ as well as the wild type mice 6–8 weeks old. Then the mice were sacrificed for collection of tissues and measuring the disease parameters^13^.

### Cell lines

LS174T, 136–7 vector only, 1049–7 vector only and LS174T-Mpi KO cell lines (136–5 Mpi KO and 1049–5 Mpi KO) were cultured in DMEM media. Media was supplemented with 10% FBS, 2mM glutamine, 100 units/L streptomycin, 100 units/L penicillin for WT cell lines whereas for LS174T-Mpi KO cell lines 10mg/ml puromycin was added. Cells were maintained in 37° C incubator with 5% CO_2_ and subcultured every 3 to 4 days in ratio of 1:3.

### Microbiome Analysis

Two pellets of the stool samples were collected from Mpi^F/F^ and Mpi^ΔVillin^ into the tubes provided by Transnetyx and sent for the shotgun whole genome sequencing, as described previously^14^. The data were analyzed using the One-Codex platform^15^of Transnetyx.

### Proteomics analysis from the colon and Small Intestine lysate

Proteomics analysis of the intestinal Tissue lysates from Colon and Small Intestine were performed as previously described^16^. Tissues from Mpi^F/F^ and Mpi^ΔVillin^ mice were collected run on (Nupage 4–12% Tris gel) (Invitrogen) for 15 mins. Then it was stained with Coomassie Brilliant Blue and then the samples were excised and sent in low retention Eppendorf tubes for Mass Spectrometry to the UTSW Proteomics Core.

### Electron Microscopy

Colon and Small Intestine tissues were collected from Mpi^F/F^ and Mpi^ΔVillin^ mice then they were fixed in with 4% paraformaldehyde, 1.5% glutaraldehyde, and 0.02% picric acid in 0.1 M cacodylate buffer at 4° C overnight before submission of the samples to the Electron Microscopy Core at UTSW. Tissues were imaged by JOEL 1400+ microscope after being mounted on grids as described previously^13^.

### Immunohistochemistry

For IHC, sections were deparaffinized and rehydrated in xylene and gradation of ethanol solutions. This was followed by Antigen Retrieval using Citrate Buffer solution at boiling temperature. The slides were washed with 1X PBS 3 times and then blocked in 1% bovine serum albumin (BSA). The primary antibodies were diluted in the same concentration of BSA in PBS and incubated overnight at 4 °C. Next the slides were washed with PBS 3X 5 mins each and incubated in secondary -HRP tagged antibody for 1 hour at room temperature as described previously^13^. Images were taken using a EVOS Microscope.

### 16sRNA FISH

16s FISH was performed as described previously^17^. Tissue sections were paraffinized and rehydrated using ethanol gradation. Then the sections were incubated in 16s RNA probes (probe sequence) conjugated with (Cy5 fluorophore) in Hybridization buffer consisting of [20 mM Tris·HCl (pH 7.4), 0.9 M NaCl, 0.1% SDS] at 50 °C overnight in a humidified chamber. Next day the slides were washed using wash buffer [20 mM Tris·HCl (pH 7.4), 0.9 M NaCl] 3X for 10 mins each. Then the sections were washed with 1X PBS 2 times. Following this the sections were processed for the MUC2 staining alongwith the nuclear staining using DAPI^17^. The images were taken using EVOS Microscope.

### Mitochondrial ETC activity measurements

An Agilent Seahorse XFe96 Analyzer was used for cellular oxygen consumption measurements of HT29-Mtx cells (Fig. 4F). Cells were plated at 20,000 cells per well in 80 μl media and allowed to adhere overnight. The following day, cells were washed twice with 200 μl per well assay medium (DMEM (Sigma-Aldrich, D5030) with 10 mM glucose, 2 mM L-glutamine, 1 mM sodium pyruvate and 1% penicillin/streptomycin), and 150 μl assay medium was added to each well after the second wash. Cells were transferred to a 37 °C, CO2-free incubator for 1 h. Standard calibration and baseline oxygen consumption measurements were performed using a 3-min ‘mix’/3-min ‘measure’ cycle with three measurements recorded at baseline and after injection of each compound. The following inhibitor was used: 5ug/ml Oligomycin, CCP, Antimycin A and 20ug/ml Tunicamycin. Data collection was performed with WAVE (v.2.4.1.1) software^18^.

### Study Approval:

All animal experiments were performed in accordance with UT Southwestern IACUC-approved protocols.

## Results

### Mpi mice are susceptible to DSS induced colitis

**Figure 1.**
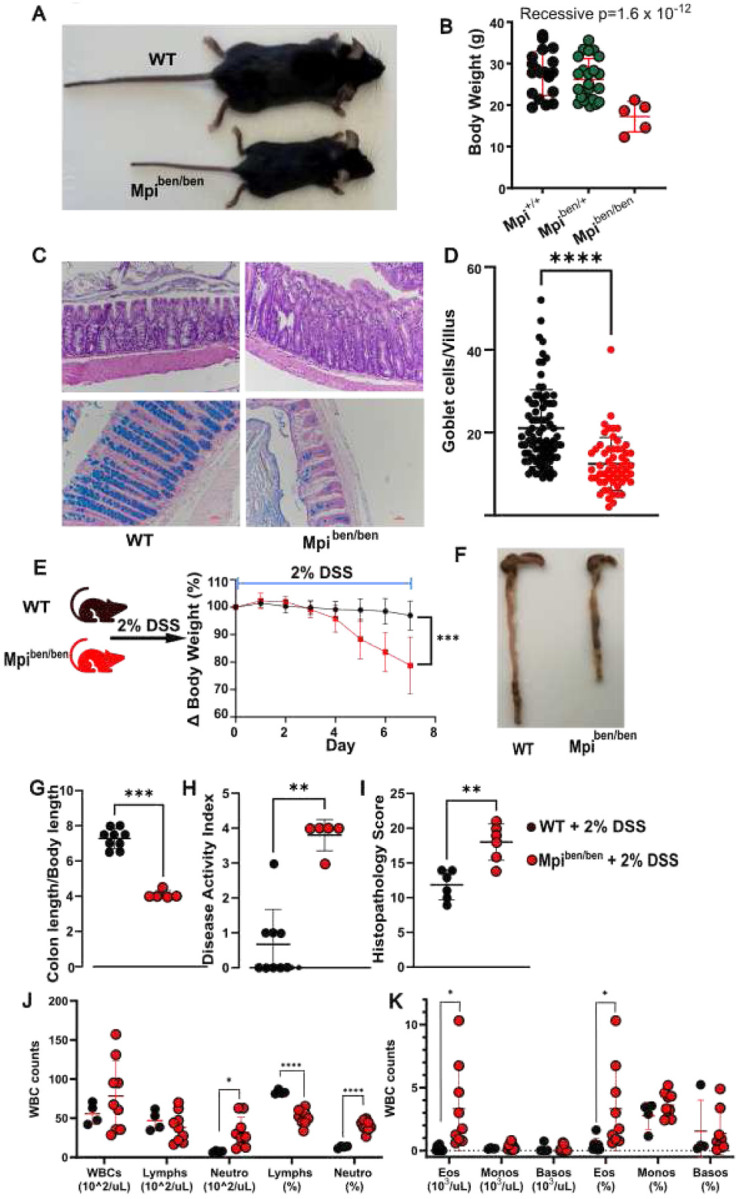
*Mpi* deficiency reduces colon Goblet cells with susceptibility to DSS-induced colitis **A,B** ENU-mutagenized Mpi^ben/ben^ mice were visibly and quantitatively smaller size compared to littermates. **C** Representative Haematoxylin and Eosin stained (top) and Alcian Blue stained (bottom) colons Mpi^+/+^ vs Mpi^ben/ben^ mice. **D** Quantification of goblet cells/Villus WT vs Benadry (villi n= 85 for Mpi^+/+^ and n=57 for Mpi^ben/ben^, across n=9 Mpi^+/+^ and n=7 Mpi^ben/ben^ mice). **E** Weight loss analysis of Mpi^+/+^ and Mpi^ben/ben^ mice after treatment with 2% DSS (n= 7 mice each group) from CRISPR Cas9 targeted mice, representative of two independent experiments. **F** Images of colon after DSS treatment for 7 days Mpi^+/+^ vs Mpi^ben/ben^. **G- I** Colon length, Disease activity index and histopathological score 7 days after DSS treatment (n= 9 for Mpi^+/+^ and n=5 for Mpi^ben/ben^). **J** Absolute and relative numbers of white blood cells (WBCs), lymphocytes (Lymphs), and neutrophils (Neutro) in peripheral blood. **K** Absolute and relative numbers of eosinophils (Eos), monocytes (Monos), and basophils (Basos) in peripheral blood. (**P<0.01, ***P<0.001, and ****P<0.0001). **D** Mann-Whitney test**, E, G, H, I** student t-test. WT= wild type, Het = heterozygote, Homo = homozygote, Dss – Dextran Sodium Sulfate. Data expressed as means ± s.d. and significance was determined as unpaired Student t-test.

During an ENU-mutagenesis forward genetic screen, a mouse strain named *benadryl* associated with a visible and allergic phenotype (Fig. 1 A,B, p=1.6×10^−12^, recessive). This strain mapped to a missense variant p.H54R in the mannose phosphate isomerase gene named *Mpi*. We have previously described this strain, which is notable for recreating most features of the human congenital disorder of glycosylation MPI-CDG including congenital diarrhea. Intestinal histopathology demonstrated a remarkable decrease of goblet cells by Alcian blue staining of glycoproteins (Fig. 1C,D, p<0.0001). This phenotype is the strongest of any reported genetic defect of goblet cells including other glycosylation related genes (Tvp23b, Yipf6) found by our group. The strength of this phenotype most closely resembles Muc2 KO, which indicates the *Mpi* gene must play an essential role in Muc2 production and goblet cell homeostasis.

Therefore, we hypothesized that *benadryl* mice would be susceptible to DSS-induced colitis. In fact, *benadryl* mice had a strong response to 2% DSS that required euthanasia by day 7 (P<0.001). Colitis was assessed by several factors, which demonstrated worse disease in *benadryl* mice. The colon length (normalized to body length) was much shorter (P<0.0005) along with an elevated disease activity index (P<0.0005) and higher histopathology score (Fig. 1 E–I). Also, fewer goblet cells lead to less mucus and higher levels of neutrophils in Mpi^ben/ben^ (Fig. 1 J–K). As defective glycosylation can contribute to protein misfolding and goblet cells are secrete high amounts of glycoproteins, we hypothesized ER stress could be exacerbated. Therefore, we measured ATF4, BiP, CHOP, and GRP78 as markers of ER stress. Every ER stress marker had elevated expression in *benadryl* mice after DSS (Fig 3B). Immunohistochemistry showed ER stress was increased across all epithelial cells, not just Goblet cells.

### Intestinal loss of Mpi causes spontaneous colitis

**Figure 2.**
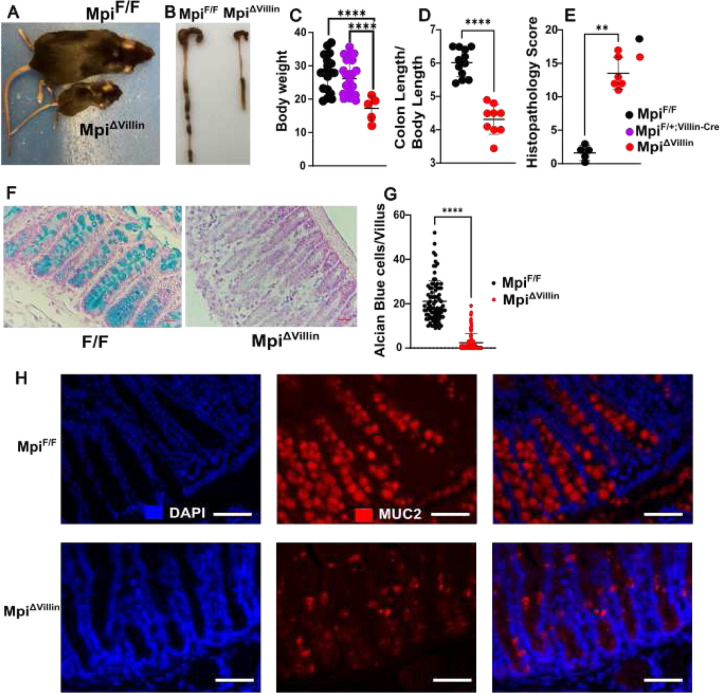
Intestinal specific Mpi knockout causes severe colon pathology. **A** Representative image for Mpi^F/F^ vs Mpi^ΔVillin^ 4 week old mice. **B** Representative image of colons from Mpi^F/F^ vs Mpi^ΔVillin^ 4 week old mice. **C** Weight of Mpi^F/F^, Mpi^F/+^ and Mpi^ΔVillin^ mice 4 weeks after birth (n= 14 for Mpi^F/F^, n= 14 for Mpi^F/+^ and n= 5 for Mpi^ΔVillin^**). D** Colonic lengths of mice 4 weeks after birth (n= 12 for Mpi^F/F^ and n= 9 for Mpi^ΔVillin^). **E** Histopathologic score of colons in Mpi^F/F^ and Mpi^ΔVillin^ mice (n=5, 6 respectively). **F** Representative Alcian Blue stained sections of colons Mpi^F/F^ vs Mpi^ΔVillin^. **G** Quantitative analysis of Goblet cells/Villus across colon for Mpi^F/F^ vs Mpi^ΔVillin^ (n=5 independent mice each group and n= 85 villi each group). **H** Representative Muc2 immunohistochemistry from Mpi^F/F^ vs Mpi^ΔVillin^ colons. **G** Mann-Whitney test **C, D** and **E** Student t-test (**P<0.01, ***P<0.001, and ****P<0.0001). ***C, D, E and G*** Data expressed as means ± s.d. and significance was determined as unpaired Student t-test. Data is representative or aggregated from at least 3 independent experiments.

**Figure 3.**
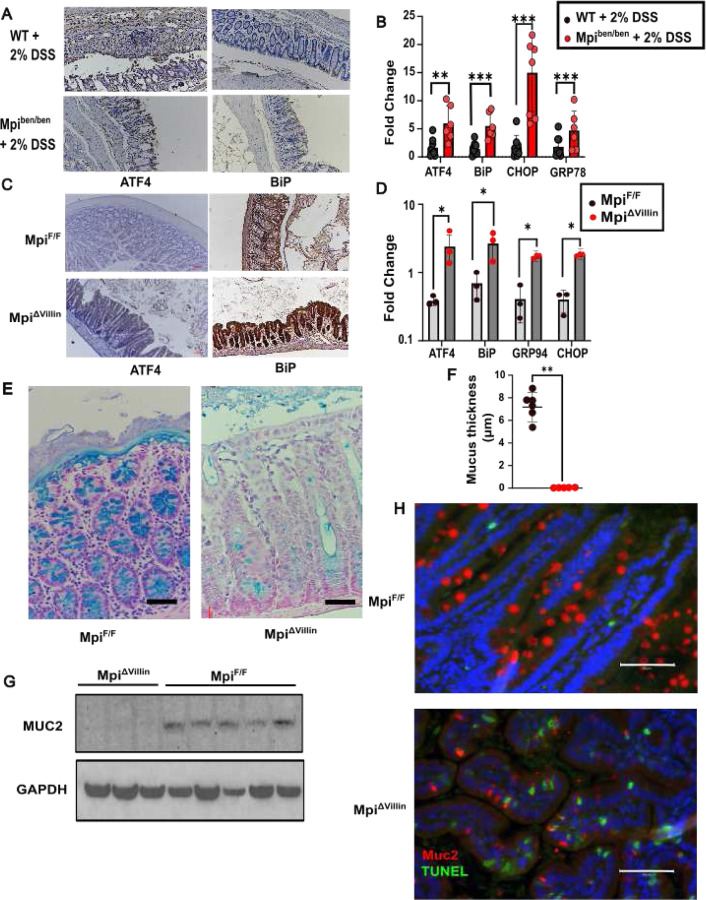
Abnormal ER stress response **A** Representative image from Immunohistochemistry for ER stress proteins ATF4 and BiP across colons of Mpi^+/+^ vs Mpi^ben/ben^ 7 days after 2% DSS treatment (repeated three times). **B** Relative mRNA expression of ER stress response specific genes- *ATF4, BiP, CHOP* and *Grp78* (n= 6 independent mice, **P<0.01, ***P<0.001). **C** Representative image from Immunohistochemistry for ER stress proteins ATF4 and BiP across colons of Mpi^F/F^ vs Mpi^ΔVillin^ (repeated three times). **D** mRNA expression of ER stress response specific genes (normalized to GAPDH, n= 3 mice each for Mpi^F/F^ and Mpi^ΔVillin^, *P<0.05). **E,F** Representative Alcian Blue stained colons and quantitative measure of mucus thickness Mpi^F/F^ vs Mpi^ΔVillin^ (repeated five times). **G** Immunoblot of mucin-2 in colon lysates from Mpi^ΔVillin^ (n=3) and Mpi^F/F^ animals (n=5) (representative image of three experiments). **H** Immunofluorescence of TUNEL (green) and Muc2 (red) from Mpi^F/F^ and Mpi^ΔVillin^ small intestine (n=3 mice each). **B,D** Data expressed as means ± s.d. and significance was determined as unpaired Student t-test. Data is representative of at least 3 independent experiments. **B, D** and **F** Student t-test. ATF4= Activating Transcription Factor 4, BiP= Immunoglobulin binding protein, CHOP= C/EBP homologous protein, Grp78= Glucose regulated protein 78, Grp94= Glucose regulated protein 94.

To test whether the colitis phenotype was intrinsic to the colon, we used an intestinal epithelial cell specific knockout (Mpi^flox/flox^; Villin^Cre^: Mpi^ΔVillin^), which lead to spontaneous colitis with a much smaller body size and shortened colon (Fig. 2 A–D)). The mice also show higher disease activity index (DAI) and histopathological score (Fig. 2E) with symptoms of rectal bleeding. The mucus layer is reduced 100 % from 8 μm to 0 μm (Fig. 3 E–F). Mucus loss is confirmed by mucin 2 (Muc2) immunohistochemistry (Fig. 2H).

### ER stress is induced by Mpi deficiency

We considered the profound loss of protective mucus could be related to protein misfolding and subsequent ER stress, which is known to impact intestinal homeostasis^19^. Muc2, is the major mucin of the intestinal mucus layer. While O-glycosylation makes up >80% of the weight of the glycoprotein, N-glycosylation has been shown to be important for subsequent dimerization and maturation in the Golgi. However, previous studies of Muc2 N-glycosylation were limited to *in vitro* studies where ER to Golgi transit was slower but maintained^20^. Cryo EM and glycopeptide mass spectrometry studies have shown Muc2 C-terminus N-glycans influence the shape of the protein as these glycans line the dimerization domain^21^. Improper glycoprotein folding can prevent ER-Golgi progression. Consequently, it appears Muc2 accumulates in the endoplasmic reticulum increasing ER stress markers of *AT4*, *BiP*, *CHOP* and *Grp94* by mRNA expression in *benadryl* mice after DSS challenge (Fig 3B) and in Mpi^ΔVillin^ at baseline (Fig 3D). The ER stress originates specifically in the epithelial cells as demonstrated by immunohistochemistry of ATF4 and BiP from DSS challenge of *benadryl* and Mpi ^ΔVillin^ mice (Fig. 3A, Fig 3C). Furthermore, we observed apoptosis of Muc2 expressing goblet cells in the small intestine, which could originate from this ER stress (Fig. 3H). The cumulative effect is higher ER stress and less mucus production for the protective mucus layer.

### N-glycosylation regulates Muc2 O-glycosylation and Goblet cell homeostasis

**Figure 4.**
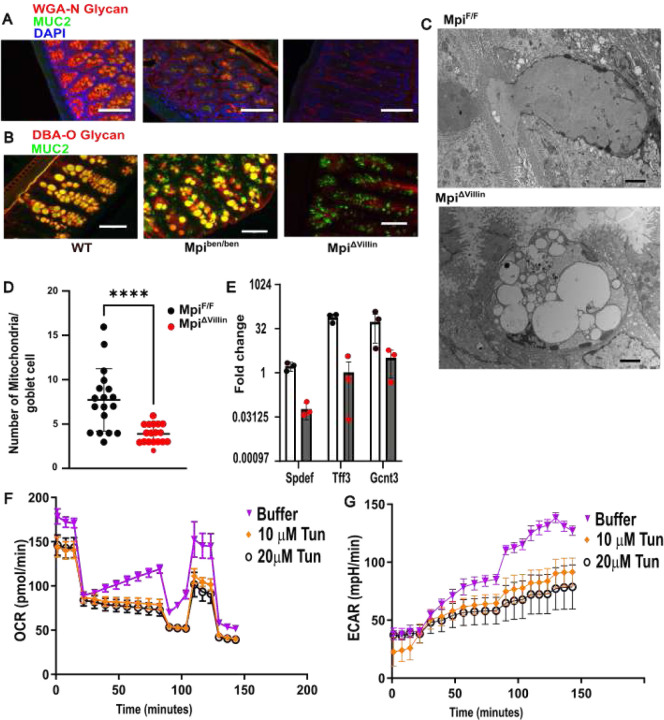
Mpi mutation affects glycosylation status that drives mucus synthesis **A** WGA and MUC2 immunofluorescent staining of Mpi^F/F^ and Mpi^F/F^; Mpi^ΔVillin^ colon tissues. **B** DBA and MUC2 immunofluorescent staining of Mpi^+/+^, Mpi^ben/ben^, and Mpi^ΔVillin^ small intestine tissues. **C** Representative electron microscopy from Mpi^F/F^ and Mpi^ΔVillin^ goblet cell (n=5 goblet cells each group from 2 different mice each group). **D** Quantitative analysis of Mitochondria/Goblet cell for Mpi^F/F^ vs Mpi^ΔVillin^ colons (n= 14 independent goblet cells from each group of mice). **E** Relative mRNA expression of Goblet cell transcription factors – *Spdef, Tff3* and *Gcnt3* (n=3 colon samples from independent mice each group). **F** Given the reduced mitochondrial mass observed on EM, mitochondrial oxygen consumption rate (OCR) and extracellular acidification rate (ECAR) were measured in HT29-MTX cells (20,000 cells/well) with deficient glycosylation (tunicamycin: 10ug/mL- 10T, 20ug/mL- 20T). (Results are the aggregate of 8–16 replicates of a single experiment). Data expressed as means ± s.d. and significance was determined as unpaired Student t-test **D** or Mann Whitney test **E**. Data is representative of at least 3 independent experiments. Spdef= SAM Pointed Domain ETS Factor, Tff3= Serum trefoil factor 3 and Gcnt3= Glucosaminyl (N-acetyl) transferase 3, WGA = Wheat Germ Agglutinin, MUC2 = mucin 2, DBA = Dolichos Biflorus Agglutinin

Since we observed less Alcian blue staining, which indicated a lower level of glycosylation in the colon, we sought to dissect the effect of Mpi deficiency on the main secreted mucin, Muc2. We first confirmed that N-glycans were decreased in the *benadryl* hypomorphic mice and then absent in Mpi^ΔVillin^ mice by using the N-glycan specific lectin WGA (wheat germ agglutinin). Similarly, Muc2 levels were decreased in *benadryl* colons with much lower levels in Mpi^ΔVillin^ (Fig 4A). Similarly, O-glycosylation, abundant on Muc2 is decreased in *benadryl* mice and absent in Mpi^ΔVillin^ (Fig 4B). Muc2 levels similarly appeared decreased by immunohistochemistry, but it was unclear if this is due to less hydration or surface area of the glycoprotein. However, western blot recapitulated a near absence of Muc2 (Fig 3G). Muc2 immunohistochemistry indicates Goblet cells are still present, but fewer in number (Fig. 2F) leading to less mucus secretion. The decreased number of Goblet cells was assessed by detection of critical transcription factors for the lineage including *Spdef, Tff3* and *Gcnt3* (Fig.4E). Each of these transcription factors were decreased by 20-fold or more, which indicates Mpi and N-glycan deficiency is deleterious to Goblet cell homeostasis. Ultrastructural analysis of Goblet cells was performed by transmission electron microscopy to evaluate sub-cellular changes. The most striking difference in Mpi^ΔVillin^ colons was the large, vacuolated Goblet cells with abnormal vesicles instead of orderly packages of mucus (Fig. 4C). These disorganized vesicles resemble lysosomes that have inadequately completed lysis. Additionally, the Mpi^ΔVillin^ Goblet cells have fewer mitochondria, which could negatively affect the metabolic fitness. In enterocytes, the length of villi was decreased, which would affect the absorptive ability of the intestines. This Goblet cell dysmorphology, mitochondrial loss, and shortened villi were also found in the small intestine.

The culmination of these cellular stressors is Goblet cells death in the small intestine. TUNEL staining was enriched in Muc2 positive small intestine Goblet cells. 55% of goblet cells were TUNEL positive in Mpi^ΔVillin^ mice vs. 0% TUNEL positive in controls (Fig. 3H). TUNEL staining was also increased in Muc2 negative enterocytes but to a much lower degree (5% Mpi^ΔVillin^ vs. 0% WT). In contrast, TUNEL staining was not increased in the colon of *benadryl* or MpiΔVillin mice.

### Intestinal loss of Mpi results in spontaneous colitis

**Figure 5.**
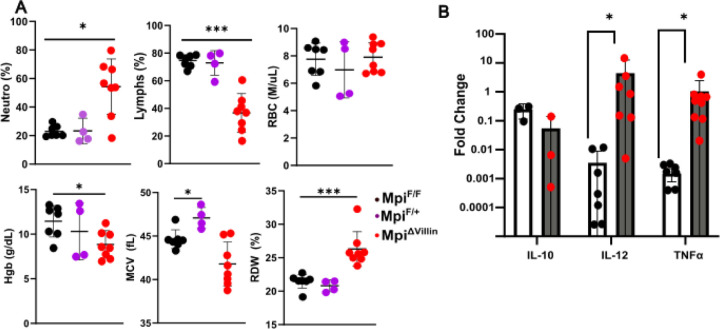
Loss of intestinal glycosylation causes Inflammation **A** Whole blood count in Mpi^F/F^, Mpi^F/+^; and Mpi^ΔVillin^ (n= 7 for Mpi^F/F^, n= 4 for Mpi^F/+^ and n= 7 for Mpi^ΔVillin^). **B** Relative mRNA expression of IL-10, IL-12 and TNFα between Mpi^F/F^ and Mpi^ΔVillin^ 4 weeks old mice (n= 7 for both Mpi^F/F^ and Mpi^ΔVillin^). Data expressed as means ± s.d. and significance was determined using One-Way ANOVA with Tukey post-hoc test for multiple comparisons **A** or Mann Whitney test **B**. Data is aggregated from 3 independent experiments.

The inflammatory nature of the colitis phenotype of Mpi^ΔVillin^ mice demonstrated by cytokine and cellular analysis. Pro-inflammatory cytokine IL-12 and TNF-alpha expression are increased 100 and 1000-fold respectively in Mpi^ΔVillin^ colons (Fig 5B), yet the anti-inflammatory cytokine IL-10 is decreased nearly 10-fold. This coincides with evidence of systemic acute inflammation with increased peripheral blood neutrophilia. The microcytic anemia with increased red cell distribution width (RDW) represents a reactive anemia, which may be related to malabsorption or acute inflammation (Fig. 1J–K).

### Mpi deficiency supports pathogenic bacteria outgrowth

**Figure 6.**
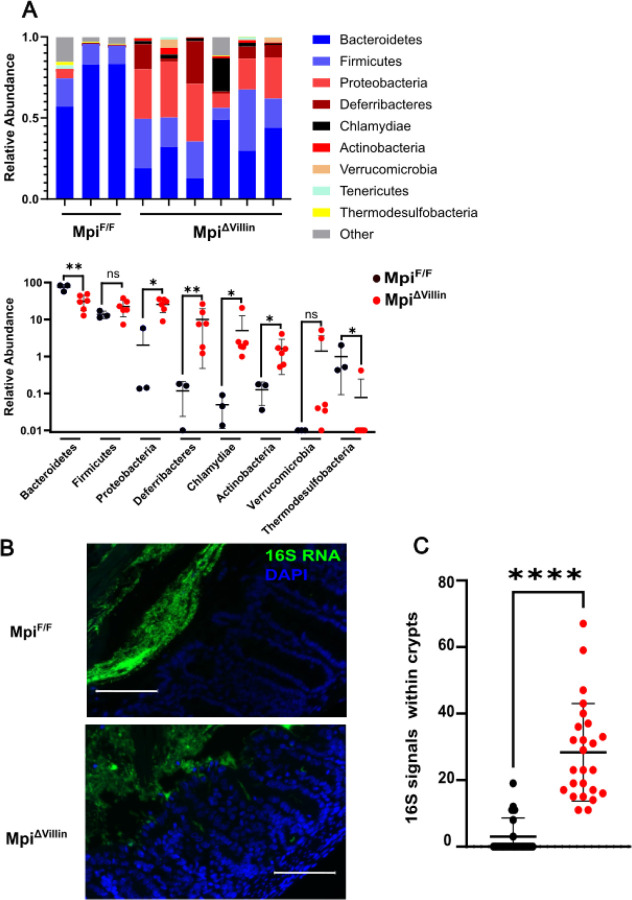
Dysbiosis and bacterial invasion. **A** Microbiome sequencing of Mpi^F/F^ and Mpi^ΔVillin^ (Update the labels to be consistent- you can mark them as 1, 2, 3 but put Mpi^F/F^ below with a line to indicate they are in the same group). (n=3 for each strain). **B** Visualization of microbiota localization relative to the small intestinal mucosal surface by 16S rRNA FISH (green) and DAPI (blue). Sections are representative of >10 littermates (n=21 villi from 3 Mpi^F/F^ mice, n= 25 villi from 5 Mpi^ΔVillin^). Scale bars=50 μm. Mice were co-housed littermates from intercrossed Mpi^ΔVillin^ mice. **C** Quantification of 16S+ signal beneath the villus tip (n=5 mice/genotype). **C** Data expressed as means ± s.d. and significance was determined by Student t-test.

Muc2 is important for maintaining the barrier between intestinal epithelium and commensal bacteria. Furthermore, mucins provide nutrition to microbes to keep them in a well-balanced equilibrium with the host. Comprising 80% of the weight of mucins, carbohydrate glycans are the nutritional source for commensal bacteria. Loss of Muc2 O-glycans secondary to N-glycan loss creates concern for dysbiosis. Dysbiosis, an imbalance in the gut microbiome, can drive disease by bacteria penetrating the epithelial barrier or secretion of altered metabolites. In Mpi^ben^ mice, the intestinal mucus layer is decreased with a nearly complete loss in Mpi^ΔVillin^ colons. 16S RNA sequencing of stools showed significant alterations in the Mpi^ΔVillin^ stools (Fig. 6A). The frequency of the symbiotic microbial phylum Bacteroidetes was 60% lower (p=0.0036). In contrast, bacteria such as Chlyamydiae, Actinobacteria, and Proteobacteria were increased (p=0.024) contributing to a state unbalanced microbiome (Fig. 6A)^22^. These changes are more than just a shift in the natural microbiome abundance but rather an outgrowth of genera known to cause disease (*Chlymadia murinatum*). Accordingly, confocal imaging of 16S FISH probe hybridized bacteria found bacterial penetration into colonic villi (Fig. 6B).

### Mpi essential for Paneth cells maturation and granulation

**Figure 7.**
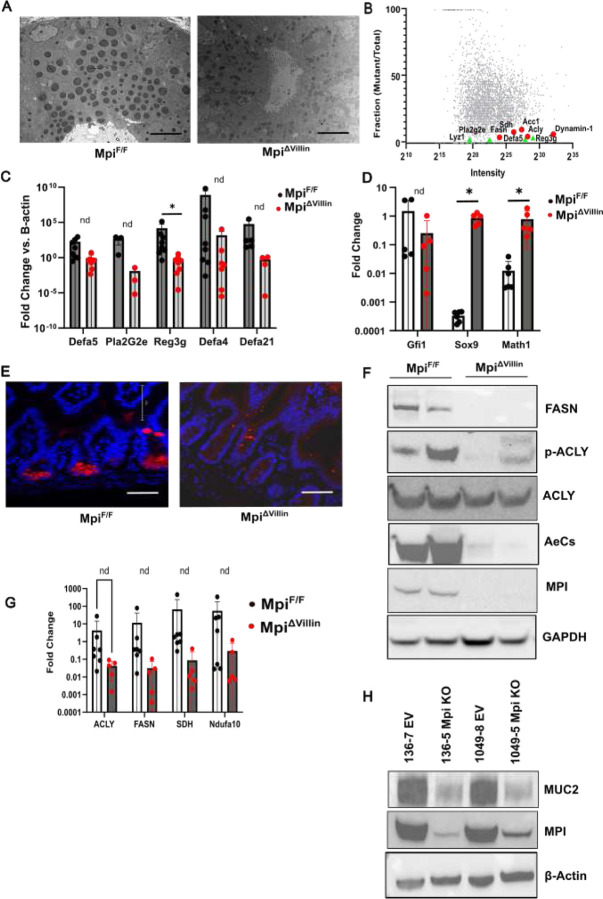
Paneth cell maturation and metabolic defects **A** Representative electron microscopy images from Mpi^F/F^ and Mpi^ΔVillin^ terminal ileum (n= 3 mice each group). **B** Composite plot of relative abundance as determined by mass spectrometry from small soluble peptide fraction of Mpi^F/F^ and Mpi^ΔVillin^ terminal ileums (n = 3 for each genotype). Normalized protein level [Mpi^ΔVillin^ sample/(Mpi^ΔVillin^ sample) + (Mpi^F/F^ sample)] is plotted on the Y-axis. Points at Y = 1 denote proteins exclusively identified in the Mpi^ΔVillin^ sample; points at Y = 0 denote proteins exclusively identified in the Mpi^F/F^ sample. Protein abundance correlates with spectral count. **C** Relative mRNA expression of Paneth cell specific genes*, Defa5= alpha defensin-5, Pla2G2e= phospholipase A2 group IIE, Reg3g= regenerating islet-derived protein 3 gamma, Defa-4= alpha defensin- 4* and *Defa21= alpha defensin- 21* (n= 8 independent ileal samples for Mpi^F/F^ and n=7 independent ileal samples for Mpi^ΔVillin^; *P<0.05). **D** Relative mRNA expression of Goblet cell specific genes, *Gfi1=Growth Factor Independent 1*, *Sox9= SRY-Box Transcription Factor 9*, and *Math1= mouse atonal homolog 1* (n= 5 independent colon samples for Mpi^F/F^ and Mpi^ΔVillin^; * P<0.05). **E** UEA-1 immunofluorescent staining for Mpi^F/F^ and Mpi^ΔVillin^ tissue. UEA-1= Ulex Europaeus Agglutinin I. **F** Immunoblot of FASN, p-ACLY, ACLY, AceCS1, MPI and GAPDH (*FASN= Fatty Acid Synthase*, *ACLY=ATP citrate lyase*, *AceCS1= Cytoplasmic Acetyl CoA Synthetase, MPI= Mannose Phosphate Isomerase* and *GAPDH= glyceraldehyde-3-phosphate dehydrogenase*) in colonocytes from 2 separate Mpi^F/F^ and Mpi^ΔVillin^ animals, representative image of 3 experiments. **G** Relative mRNA expression of metabolic specific genes, *ACLY=ATP citrate lyase*, *FASN= Fatty Acid Synthase*, *SDH= Succinate dehydrogenase* and *Ndufa10= NADH dehydrogenase [ubiquinone] 1 alpha subcomplex subunit 10* (n= 7 independent colon samples for Mpi^F/F^ and n= 5 independent colon samples for Mpi^ΔVillin^). **H** Immunoblot of MUC2, MPI and β- Actin (*MUC2= Mucin 2, MPI= Mannose Phosphate Isomerase* and *β- Actin= beta Actin*) in LS174T WT and Mpi KO cells −136–7 EV, 136–5 Mpi KO, 1049–8 EV and 1049–5 Mpi KO (where EV= Empty Vector) representative image of 3 independent experiments.

Ultrastructural analysis of the of the Small Intestine revealed loss of Paneth cell dense core granules of Mpi^ΔVillin^ compared to the wild type (Fig. 7A). Furthermore, similar to the colonic electron microscopy images, microvilli density was decreased, and Goblet cells had a similar morphology. To confirm loss of Paneth cell granules, UEA-1 lectin staining was performed and confirmed loss of signal at the base of small intestine crypts. UEA-1 binds to fucosylated glycoproteins, and less staining in the Mpi^ΔVillin^ intestines is consistent with a loss of glycosylation due to *Mpi* deletion (Fig. 7E).

Paneth cell dense granules contain a mixture of anti-microbial peptides (defensins), lysozyme, and other defensive molecules. Therefore, we used mass spectrometry to detect any change in the abundance of the small soluble molecules. We found an >95% decrease in alpha defensin 5, 20, 22, lysozyme C, and phospholipase A2 (Fig. 7B and Table 2.). We further confirmed defensin and Reg3g decreased expression of the defensins by qPCR analysis by 100–100000-fold (Fig. 7C) which complements the ultrastructural and mass spectrometry results for Mpi^ΔVillin^.

Next, we examined gene expression of factors important for differentiation of Paneth cells and the secretory progenitors. Gfi1 was unchanged, but Sox9 and Math1/Atoh1 were significantly increased by 100–1,000-fold in the Mpi^ΔVillin^ compared to wild type (Fig. 7D). This may represent a drive to create more Paneth cells since the ones present are not producing necessary defensive molecules. Overall, these results indicate that Mpi loss not only affects the glycosylated, secretory granule contents but also maturation of Paneth cells broadly.

### Mpi deficiency reduces intestinal lipid metabolism

In addition to loss of small antimicrobial peptides, mass spectrometry revealed several metabolic proteins were shifted. Many were enzymes that affected synthesis of Acyl-CoA from acetate (Aldh9a1, Accs1), citrate (Slc25A1, Acly), BH-butyrate/acetoacetate (Nnt, Bhd1), and malate/glyceraldehyde via pyruvate (malate enzyme, triose kinase). Further, enzymes essential for converting Acyl-CoA to triglycerides and fatty acids (Acc1, Fasn) were decreased. These enzymes were decreased several fold, often present at levels <10% compared to wild type. Including –*SDH* and *Ndufa10* all decreased by 10–20- fold (Fig. 7G). Proteomic findings were confirmed by western blot of ACC, FSN, and ACLY1 in Mpi^ΔVilliln^ mice (Fig. 7F). Fatty acid enzyme loss was regulated at the transcriptional level as determined by RT-qPCR analysis. Gene expression was decreased 10–100-fold compared to wild type mice (Fig. 7G). A common feature of these metabolic genes is that they are all transcriptionally regulated by SREBP1.

The large intestine was similarly found to have disrupted lipid metabolism when Mpi was conditionally deleted in epithelial cells. We subjected colon lysates to proteomic analysis. The mass spectrometry results confirmed the findings from the small intestine with a decrease in metabolic proteins including those involved in fatty acid synthesis (*Acc, Acyl1, Fasn*) and Mitochondria (*Sdh, Gpx2, cytochrome c oxidase (COX1)* and *Ndufa10*). These genes were decreased 88–96% in Mpi^ΔVilliln^ colons compared to wild type (Fig. 7B).

The decrease in mitochondrial proteins is reflected by a numerical decrease in mitochondria of Goblet cells by electron microscopy. Furthermore, Mitochondrial respiration was functionally impacted in the HT-29 MTX goblet cell line when treated with the glycosylation inhibitor tunicamycin as determined by seahorse assay (Fig. 4F). All three groups show decline in basal respiration, as expected as cells consume oxygen for energy production. Proton leak is stable across all groups during the experiment. However, there is a notable drop in ATP production in the tunicamycin treated groups, and the maximal respiration capacity is decreased. Decreased spare respiratory capacity (difference between maximal and basal respiration) indicates a diminished ability to respond to increased energy demands like during stress. Furthermore, the decreased ECAR (extracellular acidification rate) suggests tunicamycin inhibits glycolysis. Overall, these findings demonstrate the diverse metabolic effects of loss of glycosylation via Mpi deficiency.

## Discussion

This work addresses long standing questions concerning N-glycan function for mucins *in vivo*. As mucins are 80% O-glycans by weight, defects affecting these post-translational modifications were thought to be more impactful. However, we have discovered that N-glycans are even more important in Muc2 maturation as genetic deficiency of *Mpi* causing N-glycan loss leads to a phenotype as strong as Muc2 deletion. This report provides the first mechanistic insight behind congenital diarrhea and protein losing enteropathy observed in human patients with MPI-CDG. By recognizing this mechanism, we highlight potential therapeutic opportunities to support gut health by augmenting gut N-glycans.

We analyzed the intestinal phenotypes using a targeted SNP variant (Mpi^benadryl^), deletion in intestinal epithelial cells (Mpi^ΔVillin^), and inducible deletion (Mpi^ERT2-UBC-Cre^). We demonstrated that loss of N-linked glycosylation from *Mpi* deficiency creates mice susceptible to DSS-induced colitis, dysbiosis, and loss of secretory goblet and Paneth cells. Mpi is of particular importance to intestinal homeostasis through several mechanisms including loss of mucus barrier, anti-microbial peptides, ER-stress, and lipid metabolism. Mpi is so important that inducible deletion leads to wasting and death within 2–3 weeks when somatically deleted. The primary defect of mucus loss is the strongest observed from our collection of ENU-mutagenized DSS-susceptible phenotypes (Tvp23b, Yipf6, Winnie, wanna), and is comparable to Muc2 knockout. While a previous study attributed congenital diarrhea in CDGs to heparin sulfate deficiency^23^, these data indicate *Mpi* and Muc2 N-glycosylation are critical for intestinal defense and homeostasis.

While immunohistochemistry can detect residual Muc2 in Mpi^ΔVillin^ mice, remarkable lack of mucus barrier thickness, concomitant bacterial invasion, and dysbiosis. We found the residual Muc2 was no O-glycosylated, which is a mechanism for its gel-like properties that underly barrier functionality. Since >90% of glycans on Muc2 are O-glycans, the role of N-glycans has been understudied. The major functional studies were performed *in vitro* showing a slowed ER-Golgi transit time and Muc2 dimerization^20^. More recent work by the Gunner group has employed glycopeptide mass spectrometry and cryo-EM to map the C-terminal N-glycan sites. These studies showed C-terminal N-glycans regulate Muc2 dimer shape and flexibility. The mild Muc2 phenotypes observed from these *in vitro* studies, significantly underestimate the profound loss of mucus, which we found. The significance of these findings has never been evaluated *in vivo* due to a lack of viable glycosylation deficient models.

We shift the focus of glycosylation canon to N-glycans for their essential role in Muc2 maturation, subsequent O-glycosylation, and function in barrier defense. No *in vivo* study of N-glycans was previously possible due to no viable mouse model of N-glycan deficiency. Attempts to create Mpi mutants were either lethal or without phenotype. *Pmm2* mutants had significantly reduced viability and no examination of an intestinal phenotype; emerging conditional knockout models may allow for this type of study. Several CDG Type II mouse models have been created, but they are viable and affect the quality (arrangement of carbohydrates) rather than an N-glycan knockout. We use the term N-glycan KO because our previous work showed *Mpi* deficient mice had N-glycan sites without any carbohydrate at glycan sites (by glycopeptide analysis). This novel finding is supported by clinical studies of CDG Type 1, and is impactful by affecting N-glycans broadly. The ultrastructural changes of Goblet cells showed highly disordered, shrunken, and aberrant structures that could be secretory vesicles or lysosomes. Furthermore, we found fewer mitochondria. This data was corroborated by mass spectrometry data with lower number of electron transport chain proteins. Lastly, electron microscopy demonstrated fewer intestinal villous projections, which can affect absorption of nutrients. Energy balance has been shown to be essential for intestinal homeostasis from creatine kinase deficient mouse models^24^. Specifically, mitochondrial disruption can induce susceptibility to DSS colitis^25 26^.

We correlated the numerical mitochondrial defect to a functional impact on metabolism by seahorse assay in a colonic goblet cell line (HT-29MTX). Combining the ECAR and OCR data provides a more complete picture of the metabolic changes induced by tunicamycin.

The decrease in both glycolytic activity and mitochondrial respiration could come from a smaller mitochondrial mass, induced cell stress responses to preserve mitochondria, or improperly folded proteins involved in oxidative phosphorylation^27^,^28^,^29^). Glycosylation could impact mitochondrial proteins due to changes in protein folding and stability^30^) or protein targeting,^31^). Most intracellular proteins are not glycosylated, so the impact of glycosylation on mitochondrial function may be more indirect.

While examining Goblet cell ultrastructural morphology, in the small intestine, we noticed a striking decrease in Paneth cell dense granules. Unlike Goblet cells which secrete mucus, Paneth cells secrete anti-microbial peptides stored in dense granules at the base of small intestine crypts. Changes in the balance of Proteobacteria or Actinobacteria can represent a state of dysbiosis^32^. These microbes are normally increased in the small intestine, and perhaps the dual deficiency in Goblet and Paneth cells could explain the profound expansion of Proteobacteria and Actinobacteria in *Mpi* deficient mice^33 34 35^. This aligns with another recent report where Paneth cell lysozymes are decreased in an O-glycans defect secondary to Tvp23b KO. This was unexpected, because anti-microbial peptides are neither N- nor O-glycosylated. We further determined Paneth cell maturation is impaired at a transcription factor level by *Mpi* deficiency. We suspect some surface glycoprotein receptor for Paneth cell differentiation is dysfunctional due to defective glycosylation. Notch signaling regulates Atoh (Math1) and Gfi1 (Stappenbech), which our data shows increased transcription of Atoh1 and its downstream expressed gene Sox9. This would be expected to increase Paneth cells but could represent a compensatory reaction to a lack of downstream vesicle formation.

Beyond secretory protein defects, we observed defects in mitochondria and lipid metabolism that could be attributable to defective surface glycoprotein receptors. Since many surface receptors are glycosylated, lack of glycosylation could impact their folding, stability, transport or ligand binding properties. This would make sense why intracellular pathways containing mostly non-glycosylated metabolic enzymes are affected. For example, EGFR, a critical epithelial growth factor receptor, has 12 N-glycan sites described, and in vitro studies removing these sites shows this inactivates its function^36^. Hepatic dyslipidemia has been found secondary to DSS-induced microbe crossing of the barrier^37^, however our observations were made in the intestines. Similarly, lipid metabolism is broadly affected with decreases in multiple enzymes. A common feature of all decreased lipid enzymes is that they are all regulated by SREBP proteins. SREBP exits the ER to the Golgi complexed with its escort protein, SCAP. While SREBP is not glycosylated, there are 3 predicted N-glycan sites on SCAP. A previous report demonstrates N-glycosylation stabilizes SCAP by reducing its interaction with Insig-1 to facilitate SCAP/SREBP trafficking from the ER to Golgi for activation^38^. Our report therefore identifies *Mpi* as a novel therapeutic target to modulate lipogenesis, which is important for metabolism and some malignancies.

We have separately reported that mannose supplementation reverses the low weight and other phenotypes of *Mpi* deficiency. Mannose supplementation was recently linked to improving symptoms of DSS-induced colitis and IL-10 KO IBD models in mice. A study by Dong et al. attributed the action of mannose to stabilizing lysosomes, but the link was not clear. This could be a contributing factor but given the important role of glycosylation in Muc2 maturation, it is possible that mannose increases N-glycan precursors supporting the synthesis of Muc2. Mpi, like many enzymes, could act as a rate limiting step that could be bypassed by mannose. If true, we would hypothesize mannose supplementation increases Muc2 production and protection from gut microbiome dysbiosis. Increasing gut protection from natural mucus could significantly aid several IBD disorders or other conditions affected by microbiome dysbiosis.

This study has opened several new questions on the role of N-glycans for functional Muc2. Through elegant studies of glycopeptide mass spectrometry and cryo-electron microscopy (cryo-EM)^39 40^ 17 occupied N-glycan sites have been mapped to the C-terminus of MUC2^41^. Enzymatic removal of these glycans creates a change in the dimer shape. However, the specific N-glycans responsible have not been identified. Other studies of glycosylated proteins including the multimeric vWF or IgE indicate specific sites may be completely essential for glycoprotein synthesis or secretion^42 43^. Therefore, we would predict that the conserved oligomannose site is an essential N-glycan site for Muc2 stability. Whereas disulfide bridges have been a focus of previous studies of MUC2 dimerization, this work highlights the importance of N-glycan post-translational modifications^40^. Determining the impact of specific N-glycan sites will provide interesting mechanistic insight into future studies.

This report addresses several long-standing questions in the fields of mucin biology, host defense, microbiome, and congenital disorders of glycosylation. Our findings demonstrate the absolute necessity of N-glycans for intestinal mucin maturation, which is in turn critical for preventing pathogenic bacteria in the microbiome. Congenital diarrhea is cardinal feature of several congenital disorders of glycosylation (including MPI-CDG) and now has been linked to loss of mucin protection. Therefore, while mucins are heavily O-glycosylated, N-glycans have an essential role in maintaining intestinal homeostasis.

## Supplementary Material

1

## Data Availability

Data are available in the main article or the Supplementary Information files.
