# Supplementary material for: N-glycosylation enzyme Mpi is essential for mucin O-glycosylation, host-microbe homeostasis, Paneth cell defense, and metabolism
Source: Res Sq. 2025 Mar 25:rs.3.rs-6222474. Preprint. [Version 1] doi: 10.21203/rs.3.rs-6222474/v1 (PMC11975007; doi:10.21203/rs.3.rs-6222474/v1)
Supplement: 1 [file NIHPPrs6222474V1-supplement-1.pdf]

Supplementary Figure 1

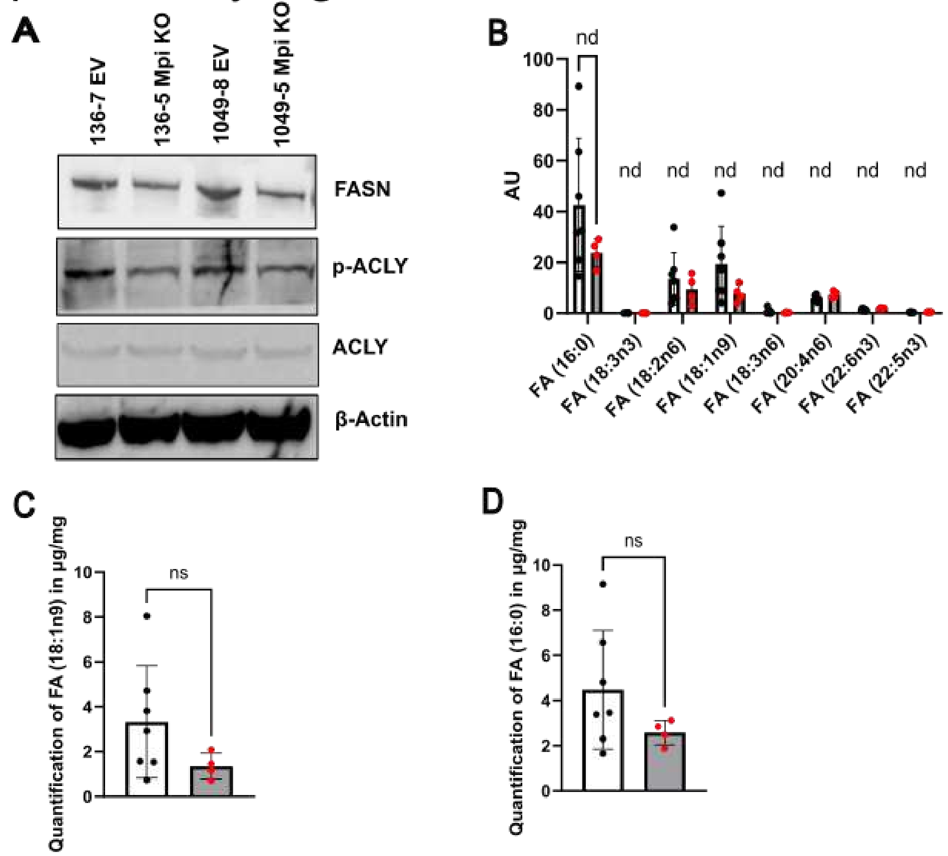

Supplementary Figure 1.

A Immunoblot of FASN, p-ACLY, ACLY and β- Actin (*FASN= Fatty Acid Synthase*, *ACLY=ATP citrate lyase* and *β-Actin= beta Actin*) in LS174T WT and Mpi KO cells -136-7 EV, 136-5 Mpi KO, 1049-8 EV and 1049-5 Mpi KO (where EV= Empty Vector) representative image of 3 independent experiments. **B** Quantitative analysis for different families of fatty acids in ileal samples from $Mpi^{F/F}$ and $Mpi^{\Delta Villin}$ mice (n= 6 for $Mpi^{F/F}$ and n= 4 for $Mpi^{\Delta Villin}$). **C, D** Quantitative analysis of FA (16:0) and FA (18:1n9) fatty acids in ileal samples from $Mpi^{F/F}$ and $Mpi^{\Delta Villin}$ (n= 7 for $Mpi^{F/F}$ and n=4 for $Mpi^{\Delta Villin}$, single experiment). Data expressed as means ± s.d. and significance was determined as Mann-Whitney test. **A, B** Data expressed as means ± s.d. and significance was determined as unpaired Student t-test.

REAGENTS or RESOURCE	SOURCE	IDENTIFIER
**Antibodies**		
Rabbit Recombinant Monoclonal MUC2	Abcam	Cat #EPR23479-47
Rabbit ATF6 Polyclonal	Proteintech	Cat# 24169-1-AP
BiP Mouse Monoclonal Antibody	Proteintech	Cat# 66574-1-Ig
Wheat Germ Agglutinin (WGA), Rhodamine (RL-1022)	Vector Laboratories	RL-1022-5
Dolichos Biflorus Agglutinin (DBA), Rhodamine (RL-1032-2)	Vector Laboratories	RL-1032-2
Ulex Europaeus Agglutinin I (UEA I), Rhodamine (RL-1062-2)	Vector Laboratories	RL-1062-2
Rabbit Monoclonal Fatty acid synthase (C20G5)	Cell Signaling Technology	Cat# 3180T
Rabbit Phospho-ATP-Citrate Lyase (Ser455)	Cell Signaling Technology	Cat# 4331T
Rabbit ATP-Citrate Lyase	Cell Signaling Technology	Cat#4332S
Rabbit Monoclonal AceCS1 (D19C6)	Cell Signaling Technology	Cat# 3658T
Mouse Monoclonal Anti-β-Actin (C4)	Santa Cruz Biotechnology	Cat# sc-47778

qRT PCR Primers list

Primer Name	Sequence
Spdef Fw	AAGGCAGCATCAGGAGCAATG
Spdef Rv	CTGTCAATGACGGGACACTG
Tff3 Fw	GGCTGCTGCTTTGACTC
Tff3 Rv	AGCCTGGACAGCTTCAA
Gcnt3 Fw	AGAGTTCCATCAACTGCTCAGG
Gcnt3 Rv	CATCCTAAGGTAGTCGGCCTC
IL-10 Fw	CAAGCCTTATCGGAAATG
IL-10 Rv	CATGGCCTTGTAGACACC
IL-12α (P35 subunit) Fw	GCCTTGGTAGCATCTATGAG
IL-12α (P35 subunit) Rv	TCGGCATTATGATTCAGAGA
TNF-α Fw	TGGCCTCCCTCTCATC

TNF-α Rv	GGCTGGCACCACTAGTT

**Chemical, peptides and recombinant proteins**	**SOURCE**	**IDENTIFIER**
Dextran Sodium Sulfate (DSS)	Thermo Fischer Scientific	Cat# J63606-22
Fetal Bovine Serum (FBS)	Rockland Immunochemicals,Inc	Cat# FBS-02-0050
DMEM (Dulbecos Modification of Eagles Medium) [+] 4.5 g/L glucose [-] L-glutamine, sodium pyruvate	Corning®	Cat#15-017-CV
Pen/Strep Cytiva	Fisher Scientific	Cat#SV30010LR1
Puromycin Dihydrochloride	Fisher Scientific	Cat#BP2956100
Sterile DMSO	Sigma-Aldrich	Cat#D2438-5X10ML
PBS 10X without calcium, magnesium	Cytiva	Cat#SH30258.01
TBST 20X, pH 7.5	Bioworld	Cat#40120065-3
Trans-Blot	Bio-Rad	Cat#1704270
**Experimental models: Cell lines**		
LS174T	This paper	
136-7 LS174T	This paper	
136-9 LS174T	This paper	
1049-8 LS174T	This paper	
1049-5 LS174T	This paper	
HT29-MTX	This paper	
**Experimental models: Organisms/Strains**		
**Mpi**$^{ben/ben}$	Mutagenix	
**C57BL/6J**	Jackson Laboratories	Cat#000664
**Mpi**$^{F/F}$	Mutagenix	
**Mpi**$^{\Delta Villin}$	Mutagenix	
